# Non-interacting, Non-opioid, and Non-barbiturate Containing Acute Medication Combinations in Headache: A Pilot Combinatorics Approach Based on DrugBank Database

**DOI:** 10.3389/fneur.2021.632830

**Published:** 2021-02-17

**Authors:** Victor Kaytser, Pengfei Zhang

**Affiliations:** Department of Neurology, Rutgers Robert Wood Johnson Medical School, New Brunswick, NJ, United States

**Keywords:** polypharmacy, drug interactions, combinatorics in medicine, big data, data science, pharmacology

## Abstract

**Background:** Polypharmacy in abortive medications is often inevitable for patients with refractory headaches.

**Objective:** We seek to enumerate an exhaustive list of headaches abortive medications that are without drug-drug interactions.

**Methods:** We updated a list of acute medications based on the widely used Jefferson Headache Manual with novel abortive medications including ubrogepant, lasmiditan, and rimegepant. Opioids and barbiturate-containing products are excluded. From this resultant list of medications, we then conducted an exhaustive search of all pair-wise interactions via DrugBank's API. Using this interaction list, we filtered all possible two, three, and four drug combinations of abortive medications. The list of medications was then reapplied to DrugBank to verify the lack of known drug-drug interactions.

**Results:** There are 192 medication combinations that do not contain any drug-drug interactions. Most common elements in these combinations are ubrogepant, prochlorperazine, followed by tizanidine. There are 67 three-drug combinations that do not contain interactions. Only two of the four-drug combinations do not yield some form of drug-drug interactions.

**Conclusion:** This list of headaches abortive medications without drug-drug interactions is a useful tool for clinicians seeking to more effectively manage refractory headaches by implementing a rational polypharmacy.

## Introduction

Non-opioid and non-barbiturate containing acute therapies are staples of headache management (1). However, excessive use of analgesics can often lead to medication overuse headaches (2). Indeed, Ferrari et al. showed that polypharmcy is present in 58.8% of those with chronic migraine (3). Therefore practitioners often use rational polypharmacy in order to manage refractory patients in real life clinical settings (4).

Although numerous articles have been published regarding medication interactions in headache medicine, we are unable to find a study conducting an exhaustive search for all medication combinations that are without interactions. We speculate that this is likely due to the heavy computational demands of achieving such an objective. However, this task is now feasible with the advent of application programming interface (API) for online databases.

The goal of this study is precisely to derive all possible combinations of abortive medications that are without interactions using DrugBank, an online pharmaceutical database (5). Our decision to use DrugBank is bore out of cautious conservatism: although DrugBank may include drug-drug interactions which are not necessarily clinically relevant, we believe that such a conservative list would provide headache providers with an accessible tool for rational polypharmacy.

## Methods

In order to include a broad list of commonly used abortive medications, we included all medications listed in the acute therapy chapter of Jefferson Headache Manual, an influential headache handbook (6). (Specifically, Tables 5.5, 5.7, 5.8, 5.14, 5.16) Since abortive medications are often used concurrently with “bridge” therapies, “bridge” medications are included in our list as well. We excluded Midrin, as it is no longer available for prescription in the United States. Since we are interested in non-opioid, non-barbiturate containing products, we also excluded butalbital and opioids. Lastly, we included lasmiditan, ubrogepant, and rimegepant; these mediations received FDA approval after the publication of the Jefferson Manual. Our full list of medications is presented in Table 1.

**Table 1:** List of abortives included and number of inclusions.

Ubrogepant	96
prochlorperazine	41
Tizanidine	37
Prednisone	31
Lasmiditan	28
Olanzapine	23
methylergonovine	17
Acetaminophen^*^	15
Aspirin^*^	8
Diflunisal	8
Fenoprofen	8
Ibuprofen^*^	8
Ketoprofen	8
meclofenamate	8
Oxaprozin	8
Salsalate	8
Sulindac	8
Tolmetin	8
Etodolac	6
Flurbiprofen	6
Indomethacin	6
Ketorolac	6
Meloxicam	6
Naproxen	6
Piroxicam	6
Droperidol	5
Naratriptan^*^	5
Diclofenac^*^	4
Nabumetone	4
Celecoxib	3
dihydroergotamine	3
Eletriptan^*^	3
Frovatriptan^*^	3
Metoclopramide^*^	3
Promethazine	3
Sumatriptan^*^	3
Zolmitriptan^*^	3
Almotriptan^*^	1
Chlorpromazine	1
Rizatriptan^*^	1
Ergotamine	0
Haloperidol	0
Dexamethasone	0
methylprednisolone	0

Asterix (*) indicates those included in Steiner et al.'s Aids to management of headache disorders in primary care (2nd edition).

Using this list, we derived all possible combinations of acute medications, paired two at a time. We then algorithmically accessed DrugBank API to determine which pairing yields a pharmacological interaction. We included all interactions, regardless of severity or clinical relevance. We call the resultant list *c*.

Using custom software, we then derived all possible combinations of abortive medications when taken two, three, and four at a time. The number of such combinations can be described mathematically by _*a*_C_2_, _*a*_C_3_, and _*a*_C_4_, respectively. Here *a* is the number of abortive medications in our list. We call each of these lists L_2_, L_3_, and L_4_, respectively.

Using list *c*, we filtered out elements of L_2_, L_3_, and L_4_ that contain any pairing of interacting medications. This produces a list of medications that are without any drug-drug interaction with respect to each other.

As a *post-hoc* analysis, we also filtered out our final resultant list of medications with evidence-based medications supplied by Table 7 and Table 8 of Steiner et al.'s *Aids to Management of Headache Disorders in Primary Care*. (Steiner et al.'s list is a superset of the one from Jefferson Manual with the exception of domperidone) (7).

Data acquisition is accomplished through Python, a programming language. Data calculations are conducted using Haskell, a functional programming language.

## Results

Data from DrugBank's API were accessed on June 6th, 2020. DrugBank did not yield a result for mefenamic acid or rimegepant. As such, these medications are removed from our study. The result is a list of 44 medications (Table 1).

We derived 946, 13,244, and 135,751 unique combinations for L_2_, L_3_, and L_4_, respectively. This is in accordance with the mathematical formula _44_C_*n*_, where *n* is 2, 3, and 4.

A total of 192 combinations of abortive medications are without any interaction. These are presented in Appendix 1. However, since any four-drug combination contains, as subsets, multiple two-drug and three-drug combinations, and any three-drug combinations contains, as subsets, multiple two-drug combinations, this list can be simplified to Table 2. In other words, each member of Table 2 contains medications that can be taken 2, 3, or 4 without interaction. (For example, consider the first item of Table 2: one can use any combinations of acetaminophen, lasmiditan, methylergonovine, and prednisone without any known interaction).

**Table 2:** Condensed list of abortive medications without interactions.

1	acetaminophen,lasmiditan,methylergonovine,prednisone
2	ubrogepant,olanzapine,methylergonovine,prednisone
3	sulindac,ubrogepant,tizanidine
4	sulindac,ubrogepant,olanzapine
5	sulindac,ubrogepant,prochlorperazine
6	tolmetin,ubrogepant,tizanidine
7	tolmetin,ubrogepant,olanzapine
8	tolmetin,ubrogepant,prochlorperazine
9	aspirin,acetaminophen,lasmiditan
10	aspirin,ubrogepant,tizanidine
11	aspirin,ubrogepant,prochlorperazine
12	zolmitriptan,ubrogepant,prednisone
13	celecoxib,ubrogepant,tizanidine
14	naratriptan,acetaminophen,prednisone
15	naratriptan,ubrogepant,prednisone
16	acetaminophen,lasmiditan,ibuprofen
17	acetaminophen,droperidol,prednisone
18	ubrogepant,frovatriptan,prednisone
19	ubrogepant,eletriptan,prednisone
20	ubrogepant,etodolac,tizanidine
21	ubrogepant,etodolac,prochlorperazine
22	ubrogepant,nabumetone,prochlorperazine
23	ubrogepant,oxaprozin,tizanidine
24	ubrogepant,oxaprozin,olanzapine
25	ubrogepant,oxaprozin,prochlorperazine
26	ubrogepant,diclofenac,prochlorperazine
27	ubrogepant,diflunisal,tizanidine
28	ubrogepant,diflunisal,olanzapine
29	ubrogepant,diflunisal,prochlorperazine
30	ubrogepant,dihydroergotamine,prednisone
31	ubrogepant,ketorolac,tizanidine
32	ubrogepant,ketorolac,prochlorperazine
33	ubrogepant,salsalate,tizanidine
34	ubrogepant,salsalate,olanzapine
35	ubrogepant,salsalate,prochlorperazine
36	ubrogepant,droperidol,prednisone
37	ubrogepant,sumatriptan,naproxen
38	ubrogepant,meloxicam,tizanidine
39	ubrogepant,meloxicam,prochlorperazine
40	ubrogepant,fenoprofen,tizanidine
41	ubrogepant,fenoprofen,olanzapine
42	ubrogepant,fenoprofen,prochlorperazine
43	ubrogepant,flurbiprofen,tizanidine
44	ubrogepant,flurbiprofen,prochlorperazine
45	ubrogepant,ibuprofen,tizanidine
46	ubrogepant,ibuprofen,prochlorperazine
47	ubrogepant,tizanidine,indomethacin
48	ubrogepant,tizanidine,meclofenamate
49	ubrogepant,tizanidine,ketoprofen
50	ubrogepant,tizanidine,piroxicam
51	ubrogepant,tizanidine,prednisone
52	ubrogepant,indomethacin,prochlorperazine
53	ubrogepant,meclofenamate,olanzapine
54	ubrogepant,meclofenamate,prochlorperazine
55	ubrogepant,olanzapine,ketoprofen
56	ubrogepant,ketoprofen,prochlorperazine
57	ubrogepant,methylergonovine,metoclopramide
58	ubrogepant,methylergonovine,promethazine
59	ubrogepant,naproxen,prochlorperazine
60	ubrogepant,piroxicam,prochlorperazine
61	ubrogepant,prednisone,prochlorperazine
62	sulindac,lasmiditan
63	tolmetin,lasmiditan
64	lasmiditan,etodolac
65	lasmiditan,nabumetone
66	lasmiditan,oxaprozin
67	lasmiditan,diclofenac
68	lasmiditan,diflunisal
69	lasmiditan,ketorolac
70	lasmiditan,salsalate
71	lasmiditan,meloxicam
72	lasmiditan,fenoprofen
73	lasmiditan,flurbiprofen
74	lasmiditan,ibuprofen
75	lasmiditan,indomethacin
76	lasmiditan,meclofenamate
77	lasmiditan,ketoprofen
78	lasmiditan,naproxen
79	lasmiditan,piroxicam
80	ubrogepant,chlorpromazine
81	ubrogepant,almotriptan
82	ubrogepant,rizatriptan

Of the list of 192, 123 (64.0%) are two-drug combinations, 67 (34.9%) are three-drug combinations, and 2 (1.0%) are four-drug combinations. Therefore, 13.0% of the all possible two-drug combinations, 0.5% of all three-drug combinations, and 0.001% of the all four-drug combinations are without interactions. Of the three-drug combinations, 59 combinations include ubrogepant. The remaining eight non-ubrogepant containing three-drug combinations include acetaminophen, prednisone, or both.

The most commonly included drugs are ubrogepant (96 inclusions), prochlorperazine (41 inclusions), tizanidine (37 inclusions), and prednisone (31 inclusions). The most commonly included medication classes are NSAIDs (133) and gepants (96 inclusions). Triptans collectively are included in only 18 combinations and 5-HT_1F_ agonists, like lasmiditan, are in 28 combinations. Haloperidol, ergotamine, methylprednisolone, and dexamethasone are in none of the non-interacting combinations.

In the *post-hoc* analysis, application of Steiner et al.'s list of abortive medication results in only three non-interacting combination of medications: (1) aspirin and acetaminophen/paracetamol, (2) naratriptan and acetaminophen/paracetamol and (3) acetaminophen and ibuprofen.

## Discussion

This is the first known instance of a “combinatoric” drug interaction study. Here, we present a list of non-opioid, non-barbiturate containing headache abortive medication combinations without any drug-drug interactions. Of course, this implies that this study also identified all medication combinations that contain a potential drug-drug interaction based on DrugBank database. Besides being an exploratory and proof of concept study, our intention is also to provide headache practitioners with a list of non-interacting and rational combination therapy.

Headache providers often use acute medications off-label. As our interest is precisely in such polypharmacy in the real-world setting, we have therefore decided to use Jefferson Headache Manual as our source of input; the manual includes a broad list of acute medications, including commonly used neuroleptics as well as muscle relaxers, such as tizanidine. As expected, this list of oral acute medications represents a superset of level A and level B evidenced medications recommended by the American Headache Society guideline as well as Steiner et al.'s *Aids to Management of Headaches* with the exception of opioids and isometheptene containing analgesics for the former and domperidone for the latter (1, 7).

### A Confirmation of Prior Critiques on Polypharmacy

Firstly, our study confirms the hypothesis that polypharmacy requires a judicious choice of medication pairing. The majority, 87% to be exact, of randomly selected two-drug combinations may produce some sort of interaction. (Indeed, if Setiner et al.'s list is to be followed, then all except three medication combinations are without interactions.) Furthermore, our study shows that randomly selecting three-drug combinations has a 99.5% chance of producing an interaction.

One reason for such a high number of drug-drug interactions is due to DrugBank's inclusion of metabolic and CPY450 interactions between medications regardless of severity of such an interaction. In other words, both pharmacodynamics and pharmacokinetics interactions are included. (Pharmacodynamics refers to interactions at levels of receptor sites where as pharmacokinetics refers to interactions in metabolism, absorption or excretion.) (8). For example, although prednisone, methylprednisolone and dexamethasone are medications of the same class, only prednisone does not interact with acetaminophen. Per DrugBank, dexamethasone can potentially increase hepatotoxicity activities of acetaminophen for dexamethasone whereas acetaminophen may increase metabolism of methylprednisolone (5). Similarly, naratriptan is the most non-interacting triptan likely due to similar reasons—for example, naratriptan does not interact with acetaminophen whereas sumatriptan does: “acetaminophen may decrease the excretion rate of sumatriptan which could result in a higher serum level.” (5).

This supports Pomes et al.'s argument that drug-drug interactions arise rather quickly even in seemingly innocent combinations and especially to those who are genetically susceptible (9). [Indeed, our project was motivated in part by Pomes et al.: “A therapeutic approach based on the personalized medicine allows to remedy similar situation by setting from the beginning of a therapy based on drug metabolically non-interfering with each other and with the functional biochemical profile of the patient…” (9). Our project simply attempts to accomplish the “metabolically non-interfering” portion for acute medication through combinatoric means; the personalized component will, of course, have to come later as modern medicine advances to take into account individual genetics profile in clinical practice (10)].

Despite our identification of 192 combinations of non-interacting medications, our position should not be construed as an encouragement for polypharmacy. Indeed, our result argues for the opposite—namely, that the majority of combinational therapy in headaches are interacting and using combination medications in a haphazard manner should be discouraged. As Martelleti suggests: “The precautionary choice of a monotherapy could minimize the risk of DDIs with subsequent AEs, and when this is not possible the physician should consult DDIs programs to ensure that the drug combination is safe.” (11). On the other hand, combinational analgesic is not by nature heretical (Steiner et al.'s list of abortive also includes a combinational analgesic from our list); should practitioners be required to use such a combination, then our article provides such a list.

Finally, we must stress that not all interactions are created equal: pharmacokinetics interactinos may only borderline increase or decrease a drug's level sublicnically. So our project is an over-estimate/upper boud for DDI rather than a lower bound.

### Which Classes Are the Most Interacting?

Specific classes of medication also present in specific patterns when multi-drug interactions are taken into account. We will detail selected medication classes in the following sections.

### NSAIDs

NSAIDs represent a commonly used class of medication for headache treatment. Our study supports its use in combination with other abortive medications. Indeed, the majority of three-drug combinations contain one NSAID. A number of NSAIDs are safe when combined with ubrogepant or with neuroleptics. It is worth noting, however, that of the only two viable four-drug combinations, no NSAID is included.

### Ubrogepant/Gepants

Ubrogepant is the most commonly occurring medication in our list and appears to be the safest medication when used in combinations. Ubrogepant combinations also comprise of the majority of viable three-drug combinations. This result, however, may be due to limited data on the gepant class due to recent release. Nonetheless, it can be argued that the addition of gepants to the migraine armamentarium may allow for the possibility of a large number of three-drug combinations.

Although rimegepant has been excluded from our search given lack of data in DrugBank, we suspect that that CGRP small molecule antagonists as a class may emerge as safe abortive options rivaling NSAIDs in the future. This is not surprising, the identification of novel antimigraine gepants as the most viable non-interacting was put forth by Pomes et al.: “These [novel agents such as gepants and ditans] not having a metabolic destiny, or rather not being subjected to enzymatic transformation or substrate of member transporters, allow to bypass the obstacles dictated by different functional biochemical setting of each individual patients and by the metabolically unfavorable drug interactions, common in the polytherapies” (9). We do, however, believe it is too premature to establish this as certain, agreeing with Martelletti, given that gepants has yet to be studied in combination: “Now that the new classes of drugs are present in the therapeutic armamentarium for migraine, such as ditans, gepants, and monoclonal antibodies for CGRPR, the need for clarity on combined therapies for migraine emerges … so, even if new drugs for the acute attack of migraine such as lasmiditan, urbrogepant, rimegepant, zavegepant will soon be available, we must use extreme caution in their use combined with previous pharmacological classes” (11).

### Triptans

Triptans rank low in Table 1, signifying that they are likely to interact with other medications when used in combinations. Therefore, triptans, surprisingly, are among the medication class with the most likely interaction when used in combination therapy. The safest triptan when used in combination is naratriptan, occurring in five combinations. We do advise cautions in the interpretation of this data as a referendum on triptan safety: DrugBank includes all theoretical interactions, a number of these that may not be seen in actual clinical practice. (As discussed in our limitation section, this is one of the major limitations to our study.) Triptans, specifically, contain a number of such interactions and therefore its risks maybe inflated in this study; an example being lack of serotonin syndrome in concomitant use of SSRI and triptans despite theoretical concerns (4). Although a detailed classification and tallying of specific kinds of interactions which produces our result would be a future direction to this study, DrugBank drug-drug interactions between sumatriptan and other abortives are shown in Table 3 in an attempt to illustrate to the reader the potential type of interaction for triptans in clinical practice when personalized medicine based on genomics are not utilized (5). As seen here a number of these interaction between NSAIDs and sumatriptan leads to increase execretion rate of sumatriptan, which may not be clinically relevant.

**Table 3:** Sumatriptan interactions.

Ubrogepant	None
Prochlorperazine	The risk or severity of adverse effects can be increased when Sumatriptan is combined with Prochlorperazine
Tizanidine	The risk or severity of adverse effects can be increased when Sumatriptan is combined with Tizanidine
Prednisone	Prednisone may decrease the excretion rate of Sumatriptan which could result in a higher serum level
Lasmiditan	The risk or severity of serotonin syndrome can be increased when Sumatriptan is combined with Lasmiditan
Olanzapine	Sumatriptan may increase the central nervous system depressant (CNS depressant) activities of Olanzapine
methylergonovine	may increase the vasoconstricting activities of Sumatriptan
Acetaminophen^*^	Acetaminophen may decrease the excretion rate of Sumatriptan which could result in a higher serum level
Aspirin^*^	Acetylsalicylic acid may decrease the excretion rate of Sumatriptan which could result in a higher serum level
Diflunisal	Diflunisal may decrease the excretion rate of Sumatriptan which could result in a higher serum level
Fenoprofen	Fenoprofen may decrease the excretion rate of Sumatriptan which could result in a higher serum level
Ibuprofen^*^	Sumatriptan may decrease the excretion rate of Ibuprofen which could result in a higher serum level
Ketoprofen	The risk or severity of adverse effects can be increased when Ketoprofen is combined with Sumatriptan
meclofenamate	may decrease the excretion rate of Sumatriptan which could result in a higher serum level
Oxaprozin	Oxaprozin may decrease the excretion rate of Sumatriptan which could result in a higher serum level
Salsalate	Salsalate may decrease the excretion rate of Sumatriptan which could result in a higher serum level
Sulindac	Sulindac may decrease the excretion rate of Sumatriptan which could result in a higher serum level
Tolmetin	Tolmetin may decrease the excretion rate of Sumatriptan which could result in a higher serum level
Etodolac	Etodolac may decrease the excretion rate of Sumatriptan which could result in a higher serum level
Flurbiprofen	Flurbiprofen may decrease the excretion rate of Sumatriptan which could result in a higher serum level
Indomethacin	Indomethacin may decrease the excretion rate of Sumatriptan which could result in a higher serum level
Ketorolac	Ketorolac may decrease the excretion rate of Sumatriptan which could result in a higher serum level
Meloxicam	Meloxicam may decrease the excretion rate of Sumatriptan which could result in a higher serum level
Naproxen	None
Piroxicam	Piroxicam may decrease the excretion rate of Sumatriptan which could result in a higher serum level
Droperidol	Droperidol may increase the central nervous system depressant (CNS depressant) activities of Sumatriptan
Naratriptan^*^	The risk or severity of adverse effects can be increased when Sumatriptan is combined with Naratriptan
Diclofenac^*^	Diclofenac may decrease the excretion rate of Sumatriptan which could result in a higher serum level
Nabumetone	Nabumetone may decrease the excretion rate of Sumatriptan which could result in a higher serum level
Celecoxib	Celecoxib may decrease the excretion rate of Sumatriptan which could result in a higher serum level
dihydroergotamine	Dihydroergocristine may increase the vasoconstricting activities of Sumatriptan
Eletriptan^*^	The risk or severity of adverse effects can be increased when Eletriptan is combined with Sumatriptan
Frovatriptan^*^	The risk or severity of adverse effects can be increased when Sumatriptan is combined with Frovatriptan
Metoclopramide^*^	The risk or severity of sedation can be increased when Sumatriptan is combined with Metoclopramide
Promethazine	Sumatriptan may increase the central nervous system depressant (CNS depressant) activities of Promethazine
Zolmitriptan^*^	The risk or severity of vasospastic reactions can be increased when Zolmitriptan is combined with Sumatriptan
Almotriptan^*^	The risk or severity of adverse effects can be increased when Sumatriptan is combined with Almotriptan
Chlorpromazine	Sumatriptan may increase the central nervous system depressant (CNS depressant) activities of Chlorpromazine
Rizatriptan^*^	The risk or severity of adverse effects can be increased when Sumatriptan is combined with Rizatriptan
Ergotamine	Ergotamine may increase the vasoconstricting activities of Sumatriptan
Haloperidol	The risk or severity of CNS depression can be increased when Haloperidol is combined with Sumatriptan
Dexamethasone	Dexamethasone may decrease the excretion rate of Sumatriptan which could result in a higher serum level
Methylprednisolone	Dexamethasone may decrease the excretion rate of Sumatriptan which could result in a higher serum level

Asterix (*) indicates those included in Steiner et al.'s Aids to management of headache disorders in primary care (2nd edition).

### Neuroleptics

Neuroleptics ranking varies widely. Prochlorperazine, for example, is the second safest when used in combination (Table 1). Olanzapine appears also to be well-tolerated when used in combination. Haloperidol, however, inevitably produces interactions when used in combination.

### Most Practical or Clinically Relevant Pairings

The most evidenced based and clinically relevant pairings supported by Steiner et al. are (1) aspirin and acetaminophen/paracetamol, (2) naratriptan and acetaminophen/paracetamol and (3) acetaminophen/paracetamol and ibuprofen. Viable three drug combinations involving these combinations of medications necessarily involves medications beyond those sanctioned by Steiner et al.: for treatments of status migrainosus, prednisone is often used as a “bridge” and can be added to naratriptan/acetaminophen combination without interactions (6). This, of course also implies that prednisone can be added to naratriptan or acetaminophen without interaction. Since naratriptan bridge could be used for treatment of patients with status migrainosus, this raises the possibility of using both a naratriptan and a prednisone bridge concurrently for status patients concurrently (12). This is not done often, to our knowledge, but is nevertheless a theoretical possibility.

On another note, lasmiditan can also be added to aspirin/acetaminophen or ibuprofen/acetaminophen combination without interactions. One should, of course, consider clinical implications of medication overuse when choosing either of these combinations.

If one is allowed (or be forced to do so by clinical circumstances) to go beyond Steiner's recommended list, then once an effective and evidenced based abortive such as NSAIDs or triptan has been identified for a patient, ubrogepant and/or tizanidine appear to be a less-interacting addition and can be added as a rescue. Should an anti-nausea medication be needed, prochlorperazine appears to be the most non-interacting. If needed, bridging therapy with prednisone appears to be non-interacting in a number of combinations. Of course, the clinician should be judicious in abortive medication's interaction with preventive medication.

### Limitations

Our study is limited by our choice of inclusion medications. Although the Jefferson Headache Manual encompasses a wide range of medications commonly used in headache medicine, it does not contain all possible medications. We believe that any of our headache colleagues will be able to show us a favorite medication that is not included in our study. However, compiling such a list is beyond the scope of our project. Indeed, our goal is an exploration of a methodological approach: we hope to show both the utility of a combinatorial approach when applied to medication interactions and to presenting a pragmatic list of useful medication combination for clinicians.

Our study is also limited by our choice of excluded medications. At the outset, we are interested only in non-opioid and non-barbiturate containing analgesics as a first assumption. While inclusion of opioid and barbiturates may broaden our project, we are unsure whether it would add any value for the result of our study; opioids and barbiturates have not been endorsed by the headache community to begin with and certainly not endorsed to be used in combination.

Our method of interrogating DrugBank does not account for severity of drug interactions. For instance, acetaminophen and ubrogepant do indeed interact, leading to potential elevation of the latter drug. However, this is in stark contrast to concurrent use of prednisone with NSAIDs, which potentiate the risk for gastrointestinal bleeding (13). In other words, having a drug interaction does not imply that the interactions are themselves serious or warrant discontinuation. There is a wide range of adverse reactions, some are more tolerable than others and severity may vary from person to person.

To overcome this limitation, a classification of specific types of interaction is needed. However, our method of parsing Drug Bank does not allow us to easily accomplish this; since interaction of medications ranges from 946 to 135,751 and DrugBank's citation of specific interactions is descriptive, manual classifications of these interactions are not possible without the helps of more advanced computational linguistic methods. We have yet to have a validated computational linguistics methods for this purpose and therefore the lack of interaction classification data remains an important limitation to our study.

Likewise, this study did not evaluate whether or not the duration of drug combination use has an effect on the risk of an adverse outcome. Most acute therapies are used in a limited capacity and we hypothesize that if a drug combination is used more frequently, then there is a higher risk of serious interaction.

### Future Directions

To better understand the possible severity of drug-drug interactions, a study compiling a list of side-effects, along with the specific drug combinations, could be useful. Previous studies have investigated the effect of headache drugs on the cytochrome P450 pathways (CYP) as a culprit for many of these interactions (3). If a four-drug combination is needed, further studies may also help to shed light on which combinations provide the most tolerable side effect risk. We can also address the effects of alcohol and other non-pharmacological interactions that may confound some of the adverse reactions experienced clinically.

The list of drugs we use in this study does not include every abortive medication and future direction can be geared toward a specialized database that compares these abortives with other non-headache medications that the patient may be taking.

Finally, acute medications in headaches are only a part of the whole management picture. To investigate the interactions between acute and prevention medication, a similar study by the Rutgers group is forthcoming.

## Conclusions

The time spent by practitioners to review and reconcile patient's medications can be cumbersome. A system to carefully address interactions between prescribed medicines can greatly reduce the medication reconciliation time and improve headache treatment. As discussed above, our project is only half of the program suggested by Pomes et al. Personalized genetics medicine may allow us to further explore how to use non-interacting combinational analgesics for specific individuals in the future. However, in an era where this is not feasible or alternatively in a society where economical, social, or political policies makes individualized genetics medicine undesirable—for example, exposures of patients to diagnosis of pre-existing conditions, economical limitations due to cost, or simply privacy concerns—our project and its methodology may provide an easily accessible list of abortive medications and their potential interactions for clinicians to readily and effectively manage their refractory headache patients.

## Data Availability Statement

The raw data supporting the conclusions of this article will be made available by the authors, without undue reservation.

## Author Contributions

PZ: conception and design and acquisition of data. PZ and VK: analysis and interpretation of data, drafting and manuscript, revising it for intellectual content, and final approval of the completed manuscript.

## Conflict of Interest

PZ has received honorarium from Alder Biopharmaceuticals, Board Vitals, Fieve Clinical Research. He collaborates with Headache Science Incorporated without receiving financial support. Ownership interest in Cymbeline LLC. The remaining author declares that the research was conducted in the absence of any commercial or financial relationships that could be construed as a potential conflict of interest.

## Abbreviations

API: Application Programming Interface
NSAIDs: Non-steroidal Anti-inflammatory Drugs
FDA: Food and Drug Administration
5HT1F: Serotonin receptor 1 F
CGRP: calcitonin gene-related peptide
CYP: cytochromes P450.
