# Supplementary material for: Non-interacting, Non-opioid, and Non-barbiturate Containing Acute Medication Combinations in Headache: A Pilot Combinatorics Approach Based on DrugBank Database
Source: Front Neurol. 2021 Feb 17;12:632830. doi: 10.3389/fneur.2021.632830 (PMC7925628; doi:10.3389/fneur.2021.632830)
Supplement: Supplementary file 1 [file Data_Sheet_1.docx]

**Appendix 1:**

List of abortive medications without interactions.

1. acetaminophen,lasmiditan,methylergonovine,prednisone,
2. ubrogepant,olanzapine,methylergonovine,prednisone,
3. sulindac,ubrogepant,tizanidine,
4. sulindac,ubrogepant,olanzapine,
5. sulindac,ubrogepant,prochlorperazine,
6. tolmetin,ubrogepant,tizanidine,
7. tolmetin,ubrogepant,olanzapine,
8. tolmetin,ubrogepant,prochlorperazine,
9. aspirin,acetaminophen,lasmiditan,
10. aspirin,ubrogepant,tizanidine,
11. aspirin,ubrogepant,prochlorperazine,
12. zolmitriptan,ubrogepant,prednisone,
13. celecoxib,ubrogepant,tizanidine,
14. naratriptan,acetaminophen,prednisone,
15. naratriptan,ubrogepant,prednisone,
16. acetaminophen,lasmiditan,ibuprofen,
17. acetaminophen,lasmiditan,methylergonovine,
18. acetaminophen,lasmiditan,prednisone,
19. acetaminophen,droperidol,prednisone,
20. acetaminophen,methylergonovine,prednisone,
21. lasmiditan,methylergonovine,prednisone,
22. ubrogepant,frovatriptan,prednisone,
23. ubrogepant,eletriptan,prednisone,
24. ubrogepant,etodolac,tizanidine,
25. ubrogepant,etodolac,prochlorperazine,
26. ubrogepant,nabumetone,prochlorperazine,
27. ubrogepant,oxaprozin,tizanidine,
28. ubrogepant,oxaprozin,olanzapine,
29. ubrogepant,oxaprozin,prochlorperazine,
30. ubrogepant,diclofenac,prochlorperazine,
31. ubrogepant,diflunisal,tizanidine,
32. ubrogepant,diflunisal,olanzapine,
33. ubrogepant,diflunisal,prochlorperazine,
34. ubrogepant,dihydroergotamine,prednisone,
35. ubrogepant,ketorolac,tizanidine,
36. ubrogepant,ketorolac,prochlorperazine,
37. ubrogepant,salsalate,tizanidine,
38. ubrogepant,salsalate,olanzapine,
39. ubrogepant,salsalate,prochlorperazine,
40. ubrogepant,droperidol,prednisone,
41. ubrogepant,sumatriptan,naproxen,
42. ubrogepant,meloxicam,tizanidine,
43. ubrogepant,meloxicam,prochlorperazine,
44. ubrogepant,fenoprofen,tizanidine,
45. ubrogepant,fenoprofen,olanzapine,
46. ubrogepant,fenoprofen,prochlorperazine,
47. ubrogepant,flurbiprofen,tizanidine,
48. ubrogepant,flurbiprofen,prochlorperazine,
49. ubrogepant,ibuprofen,tizanidine,
50. ubrogepant,ibuprofen,prochlorperazine,
51. ubrogepant,tizanidine,indomethacin,
52. ubrogepant,tizanidine,meclofenamate,
53. ubrogepant,tizanidine,ketoprofen,
54. ubrogepant,tizanidine,piroxicam,
55. ubrogepant,tizanidine,prednisone,
56. ubrogepant,indomethacin,prochlorperazine,
57. ubrogepant,meclofenamate,olanzapine,
58. ubrogepant,meclofenamate,prochlorperazine,
59. ubrogepant,olanzapine,ketoprofen,
60. ubrogepant,olanzapine,methylergonovine,
61. ubrogepant,olanzapine,prednisone,
62. ubrogepant,ketoprofen,prochlorperazine,
63. ubrogepant,methylergonovine,metoclopramide,
64. ubrogepant,methylergonovine,prednisone,
65. ubrogepant,methylergonovine,promethazine,
66. ubrogepant,naproxen,prochlorperazine,
67. ubrogepant,piroxicam,prochlorperazine,
68. ubrogepant,prednisone,prochlorperazine,
69. olanzapine,methylergonovine,prednisone,
70. sulindac,lasmiditan,
71. sulindac,ubrogepant,
72. sulindac,tizanidine,
73. sulindac,olanzapine,
74. sulindac,prochlorperazine,
75. tolmetin,lasmiditan,
76. tolmetin,ubrogepant,
77. tolmetin,tizanidine,
78. tolmetin,olanzapine,
79. tolmetin,prochlorperazine,
80. aspirin,acetaminophen,
81. aspirin,lasmiditan,
82. aspirin,ubrogepant,
83. aspirin,tizanidine,
84. aspirin,prochlorperazine,
85. zolmitriptan,ubrogepant,
86. zolmitriptan,prednisone,
87. celecoxib,ubrogepant,
88. celecoxib,tizanidine,
89. naratriptan,acetaminophen,
90. naratriptan,ubrogepant,
91. naratriptan,prednisone,
92. acetaminophen,lasmiditan,
93. acetaminophen,droperidol,
94. acetaminophen,ibuprofen,
95. acetaminophen,methylergonovine,
96. acetaminophen,prednisone,
97. lasmiditan,etodolac,
98. lasmiditan,nabumetone,
99. lasmiditan,oxaprozin,
100. lasmiditan,diclofenac,
101. lasmiditan,diflunisal,
102. lasmiditan,ketorolac,
103. lasmiditan,salsalate,
104. lasmiditan,meloxicam,
105. lasmiditan,fenoprofen,
106. lasmiditan,flurbiprofen,
107. lasmiditan,ibuprofen,
108. lasmiditan,indomethacin,
109. lasmiditan,meclofenamate,
110. lasmiditan,ketoprofen,
111. lasmiditan,methylergonovine,
112. lasmiditan,naproxen,
113. lasmiditan,piroxicam,
114. lasmiditan,prednisone,
115. ubrogepant,frovatriptan,
116. ubrogepant,eletriptan,
117. ubrogepant,chlorpromazine,
118. ubrogepant,etodolac,
119. ubrogepant,almotriptan,
120. ubrogepant,nabumetone,
121. ubrogepant,oxaprozin,
122. ubrogepant,diclofenac,
123. ubrogepant,diflunisal,
124. ubrogepant,dihydroergotamine,
125. ubrogepant,ketorolac,
126. ubrogepant,salsalate,
127. ubrogepant,droperidol,
128. ubrogepant,sumatriptan,
129. ubrogepant,meloxicam,
130. ubrogepant,fenoprofen,
131. ubrogepant,flurbiprofen,
132. ubrogepant,ibuprofen,
133. ubrogepant,tizanidine,
134. ubrogepant,indomethacin,
135. ubrogepant,meclofenamate,
136. ubrogepant,olanzapine,
137. ubrogepant,ketoprofen,
138. ubrogepant,methylergonovine,
139. ubrogepant,metoclopramide,
140. ubrogepant,naproxen,
141. ubrogepant,piroxicam,
142. ubrogepant,prednisone,
143. ubrogepant,prochlorperazine,
144. ubrogepant,promethazine,
145. ubrogepant,rizatriptan,
146. frovatriptan,prednisone,
147. eletriptan,prednisone,
148. etodolac,tizanidine,
149. etodolac,prochlorperazine,
150. nabumetone,prochlorperazine,
151. oxaprozin,tizanidine,
152. oxaprozin,olanzapine,
153. oxaprozin,prochlorperazine,
154. diclofenac,prochlorperazine,
155. diflunisal,tizanidine,
156. diflunisal,olanzapine,
157. diflunisal,prochlorperazine,
158. dihydroergotamine,prednisone,
159. ketorolac,tizanidine,
160. ketorolac,prochlorperazine,
161. salsalate,tizanidine,
162. salsalate,olanzapine,
163. salsalate,prochlorperazine,
164. droperidol,prednisone,
165. sumatriptan,naproxen,
166. meloxicam,tizanidine,
167. meloxicam,prochlorperazine,
168. fenoprofen,tizanidine,
169. fenoprofen,olanzapine,
170. fenoprofen,prochlorperazine,
171. flurbiprofen,tizanidine,
172. flurbiprofen,prochlorperazine,
173. ibuprofen,tizanidine,
174. ibuprofen,prochlorperazine,
175. tizanidine,indomethacin,
176. tizanidine,meclofenamate,
177. tizanidine,ketoprofen,
178. tizanidine,piroxicam,
179. tizanidine,prednisone,
180. indomethacin,prochlorperazine,
181. meclofenamate,olanzapine,
182. meclofenamate,prochlorperazine,
183. olanzapine,ketoprofen,
184. olanzapine,methylergonovine,
185. olanzapine,prednisone,
186. ketoprofen,prochlorperazine,
187. methylergonovine,metoclopramide,
188. methylergonovine,prednisone,
189. methylergonovine,promethazine,
190. naproxen,prochlorperazine,
191. piroxicam,prochlorperazine,
192. prednisone,prochlorperazine,
